# Grubbs’ and Schrock’s Catalysts, Ring Opening Metathesis Polymerization and Molecular Brushes—Synthesis, Characterization, Properties and Applications

**DOI:** 10.3390/polym11020298

**Published:** 2019-02-11

**Authors:** Ioannis Choinopoulos

**Affiliations:** Department of Chemistry, Industrial Chemistry Laboratory, National and Kapodistrian University of Athens, Panepistimiopolis Zografou, 15771 Athens, Greece; ichoinop@chem.uoa.gr

**Keywords:** molecular brush, bottlebrush, Ring Opening Metathesis Polymerization (ROMP), macromonomer, polynorbornene, Grubbs’ catalysts, poly(ethylene oxide), polylactide, poly(ε-caprolactone), poly(methacrylate)

## Abstract

In this review, molecular brushes and other macromolecular architectures bearing a bottlebrush segment where the main chain is synthesized by ring opening metathesis polymerization (ROMP) mediated by Mo or Ru metal complexes are considered. A brief review of metathesis and ROMP is presented in order to understand the problems and the solutions provided through the years. The synthetic strategies towards bottlebrush copolymers are demonstrated and each one discussed separately. The initiators/catalysts for the synthesis of the backbone with ROMP are discussed. Syntheses of molecular brushes are presented. The most interesting properties of the bottlebrushes are detailed. Finally, the applications studied by different groups are presented.

## 1. Introduction

In 2005, Yves Chauvin, Robert H. Grubbs and Richard R. Schrock shared the Nobel award in chemistry on their work regarding the development of the metathesis method in organic synthesis [1]. Metathesis is a word borrowed from the Greek language (μετάθεσις). Its common meaning is changing and in our case it refers to the rearrangement of the molecular fragments. This reaction was discovered during oligomerization of ethylene in the 1950s [2]. Not surprisingly, it has been utilized in the synthesis of polymers by various methods. One of those polymerization methods is ring opening metathesis polymerization (ROMP), a term first used by Tim Swager when he was a graduate student in R. H. Grubbs’ laboratory at the California Institute of Technology (Caltech) [3].

**Figure 1:**
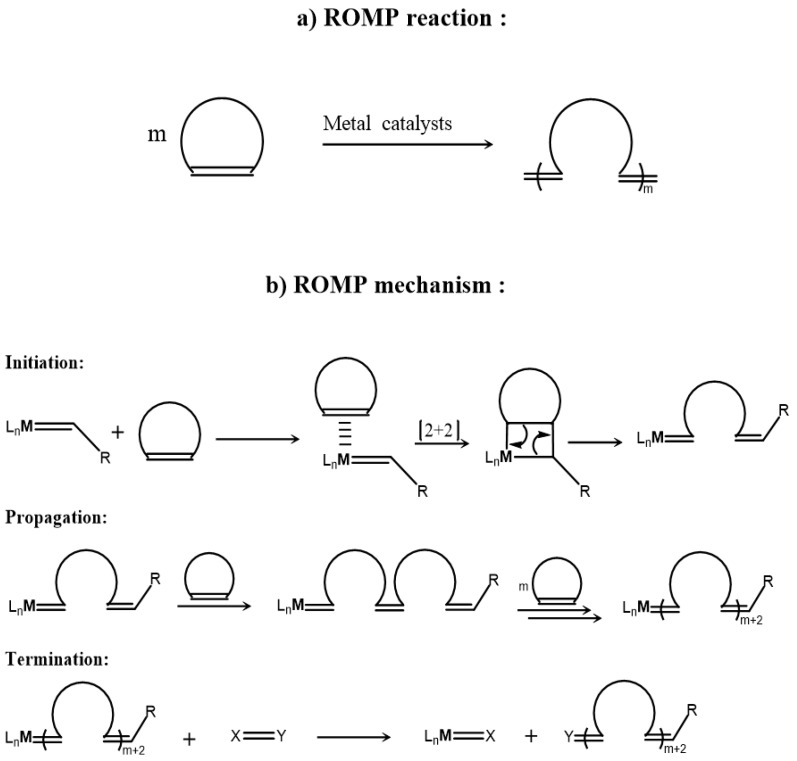
(**a**) ROMP reaction of cyclic olefins (double bonds along the main chain). (**b**) Simplified general mechanism of ROMP reaction of cyclic olefins.

ROMP is a special type of polymerization, where the unsaturated ring of a cyclic olefin opens and the number of unsaturated bonds is retained in the polymer backbone. This is contradictory with the classic polymerization of alkenes, where the main chain consists of single carbon–carbon bonds (Figure 1). The driving force for ROMP is the ring strain of the cyclic monomer, but a metal initiator/catalyst is required for the polymerization to occur.

**Figure 2:**
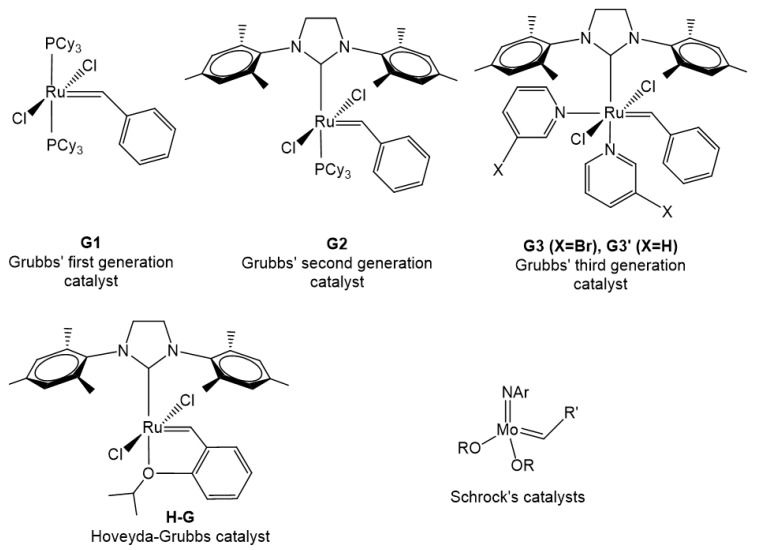
Grubbs’ and Schrock’s catalysts.

The initial ROMP reactions were performed employing undefined catalytic systems, like TiCl_4_/Et_3_Al, MoCl_5_/Et_3_Al, WCl_6_/EtAlCl_2_/EtOH, Re_2_O_7_/Al_2_O_3_, RuCl_3_/polar solvents and many more [3]. Since the first discovery of metathesis and ROMP, an enormous effort has taken place in order to understand the mechanism and develop new initiators/catalysts suitable for each emerging case. Nowadays, the majority of drawbacks has been overcome by well-defined transition metal complexes, some of which are commercially available (Schrock, Grubbs, Hoveyda catalysts). Although, Schrock’s catalysts were mainly employed in the initial efforts for bottlebrush synthesis [4,5,6,7,8], the discovery and commercial availability of Grubbs’ catalysts rendered ring opening metathesis polymerization (ROMP) a useful tool for the synthesis of bottlebrush copolymers (Figure 2). The major problems of catalyst functional group tolerance, sensitivity towards air and moisture and degree of polymerization (DP) of macromonomers were solved by Grubbs’ catalysts. Due to its high activity and functional group tolerance, Grubbs’ third generation catalyst (**G3** and **G3’**) is commonly used in bottlebrush synthetic procedures in our days.

**Figure 3:**
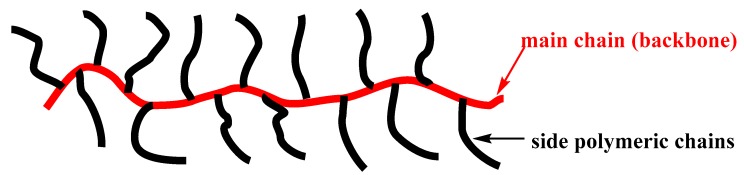
Molecular brush structure.

A molecular brush is a grafted (co)polymer that consists of a linear main polymeric chain (backbone) and side polymeric chains (Figure 3). The reader should not confuse polymeric brushes, which refer to linear polymers attached to surfaces, with molecular brushes. The unique feature of this complex macromolecular architecture is that every repeating unit of the backbone bears at least one side polymeric chain (densely grafted copolymers). Due to their topology, they are referred to as molecular brushes, cylindrical brushes, bottlebrushes or polymacromonomers (when they derive from “graft through” polymerization method). ROMP is employed to synthesize the main chain and (oxa)norbornene macromonomers or (oxa)norbornene derivatives are mainly used. A few examples where the main chain is formed by ROMP of cyclobutene macromonomers also exist [9,10,11,12,13]. This review deals only with macromolecules which are pure molecular brushes, or bear a molecular brush fragment, and their backbone was synthesized by ROMP mediated by Mo or Ru metal complexes. Meaning that, not densely grafted (co)polymers, as well as molecular brushes which main chain was synthesized with methods other than ROMP, will not be considered.

**Figure 4:**
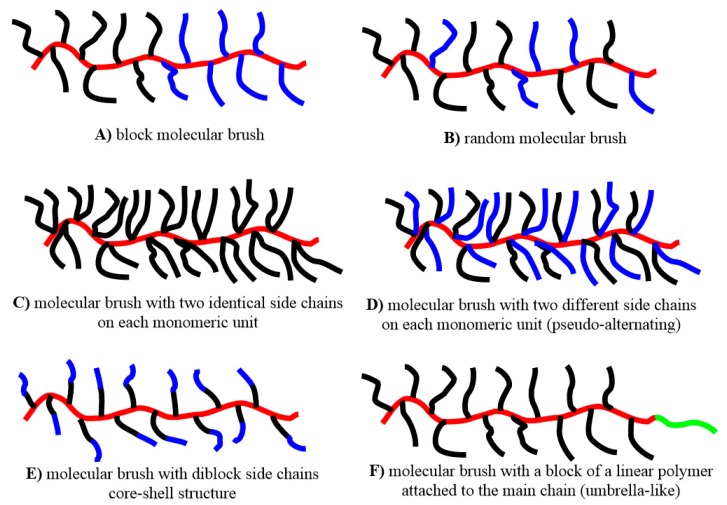
A few advanced macromolecular architectures of molecular brushes.

Advanced macromolecular architectures of molecular brushes have been synthesized too (Figure 4). Since there can be a variety of side chains, synthesizing molecular brushes with more than one type of side chains leads to molecular brushes with blocks (Figure 4A) or a random distribution (Figure 4B) of side chains. It is possible for each backbone monomeric unit to bear two side polymeric chains, of the same (Figure 4C) or different (pseudo-alternating, Figure 4D) nature. The second side chain might be attached to the norbornene (NBE) moiety or both chains can be attached to a suitable spacer fragment. The result is more densely grafted architectures. Instead of a second polymeric chain, a small molecule can be attached, depending on the application. Small molecules can also be attached at the end of the bottlebrush main chain as side groups of a norbornene monomer (Figure 4F). The end of the backbone can also be capped with a suitable reagent. The carbon–carbon double bonds of the main chain and the functional end groups of the side chains can also be exploited in post-polymerization reactions. Core–shell bottlebrushes (Figure 4E) consist of diblock copolymer side chains, possessing intriguing properties in each single molecule. More complex architectures, like star architectures bearing bottlebrush arms can be synthesized too. 

Even though most of the bottlebrush properties derive from the nature and features of the polymeric side chains, the unusual architecture provides a number of unique and potentially useful properties as well. Novel or enhanced features, compared to linear (co)polymers, are attributed to the complex macromolecular architecture of a molecular brush. Molecular brushes show characteristic spherical, cylindrical or worm-like conformations as a result of intramolecular interactions amongst the densely grafted side chains. This is a feature of the low entanglement of the side chains (morphology features of specific architectures are mentioned in Section 6). The ease of adjustment of the main and side chains renders the fine tuning of each property quite easy. Depending on the application, the nature and the appropriate length of the side chains are chosen. For example, like linear block copolymers, amphiphilic bottlebrushes with blocks of different side chains (hydrophilic, hydrophobic) can be synthesized.

The possible combinations of side chains and/or small molecules are literally endless, but limitations are imposed by the reaction chemistry. Nevertheless, there are a lot of unexplored territories in molecular brush synthesis and the arising properties and applications.

## 2. History—From Propylene Metathesis to Molecular Brushes

**Figure 5:**
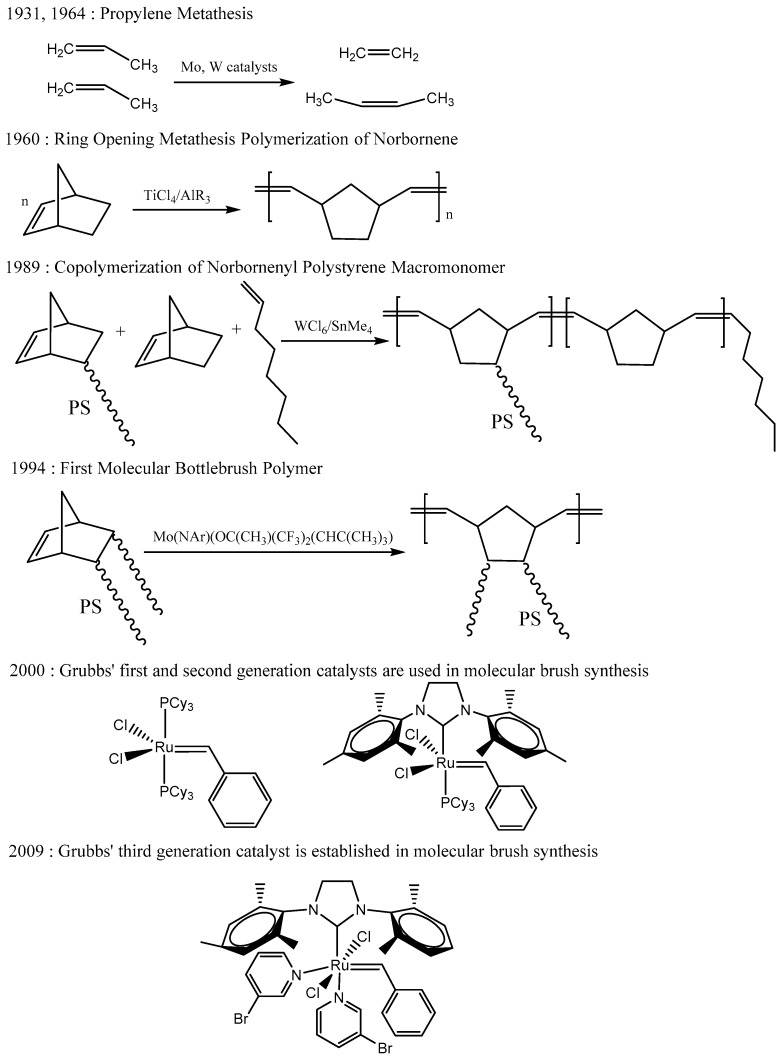
Milestones in the history of ROMP and molecular brush synthesis.

This historic reference contains the most critical discoveries, which led to the establishment of Grubbs’ catalysts as almost the absolute tools for the synthesis of bottlebrush macromolecular architectures using ROMP nowadays. Other reviews discussing generally the metathesis reaction contain more or less the same main historic points, as well as a few others regarding each writer’s point of view (Figure 5). It must be mentioned that graft copolymers where the main chain was synthesized with the random incorporation of a small monomer were not taken under consideration, since the synthesis of densely grafted bottlebrush copolymers exhibited different experimental problems, some of which were overcome with the incorporation of a small monomer.

In 1931, metathesis of propylene into ethylene and butylene was observed. Unfortunately, the scope of that project was not the, unknown then, metathesis reaction and no further research was conducted [14]. Two decades later (1954), an unsaturated polynorbornene was synthesized [15]. In 1964, the metathesis of propylene was studied with molybdenum and tungsten catalysts [16]. Two more important discoveries, the catalytic ring opening polymerization of cyclopentene and the double bond between a metal and carbon (metallocarbene) were the reason why 1964 was characterized as a “magic year” [17,18]. As far as ROMP is concerned, the polymerization of norbornene by the binary systems WCl_6_/AlEt_2_Cl or TiCl_4_/AlR_3_ was studied in 1960 [19,20]. Seven years later (1967), it was realized that the ring opening polymerization of cyclic alkenes and the disproportionation of acyclic alkenes were the same type of reaction, the metathesis reaction [21]. The mechanism of the metathesis reaction was proposed correctly in 1971 [22].

To my knowledge, the first time a macromonomer was utilized in ROMP reactions was in 1989. *ω*-norbornenyl polystyrene macromonomers were synthesized and used to prepare poly(norbornene-*g*-styrene) employing the WCl_6_/SnMe_4_ system. This copolymer was not a bottlebrush copolymer, since norbornene was copolymerized with the macromonomers [23]. The first bottlebrush polymacromonomer bearing a polynorbornene (PNBE) main chain synthesized by ROMP was prepared in 1994. A Schrock molybdenum catalyst, Mo(NAr)(OC(CH_3_)(CF_3_)_2_(CHC(CH_3_)_3_), was used and well-characterized polymers formed, but low degrees of polymerization (DP) were achieved (<10) and macromonomers with longer chains produced bimodal polydispersities [4]. Since experimenting with sensitive systems requires experienced researchers and fine tuning of the polymerization reaction in order to prepare the desired bottlebrush structure, the work with Schrock’s catalysts (Mo) was limited [4,5,6,7,8,9,24,25,26,27,28,29,30]. The idea to prepare bottlebrush copolymers bearing blocks of different side chains and the idea to prepare bottlebrush copolymers bearing side chains consisting of different blocks were studied with molybdenum catalysts [26,27,28,29]. Another aspect was to prepare bottlebrush copolymers bearing two side chains in each monomeric unit, which was originally studied using molybdenum catalysts in 1997–1998 [24,25].

In 1998, a ruthenium carbene for bottlebrush synthesis was prepared in situ with the [RuCl_2_(*p*-cymene)]_2_/tricyclohexylphosphine (PCy_3_)/(trimethylsilyl)diazomethane system. The poly(norbornene)-g-poly(ε-caprolactone) bottlebrush copolymers were synthesized employing “graft from” and “graft through” methods. The results were not very encouraging, since low degrees of polymerization were achieved using the “graft through” method [31,32]. The same year, another ruthenium carbene, [RuCl_2_(PPh_3_)_2_CHCHCPh_2_], was used with little success [30]. In 2000, the use of Grubbs’ first (**G1**) and second (**G2**) generation catalysts for the preparation of “small” molecular brushes was published [33]. Even though the side chains were oligomers of amino acids and ethylene oxide, I consider this the initiation of employing Grubbs’ catalysts for the synthesis of molecular brushes. Four years later, the synthesis of ultralarge poly(*l*-lactide) molecular brushes exhibited one very important key feature of Grubbs’ catalysts [34]. In 2009, **G3** (and **G3’**) was exploited in the synthesis of molecular brushes [35,36,37,38]. The amazing results encouraged more researchers to employ mainly pyridine modified **G3’** and quite less **G3** as irreplaceable tools for bottlebrush synthesis with ROMP since then.

## 3. Macromolecular Brush Synthetic Strategies

**Figure 6:**
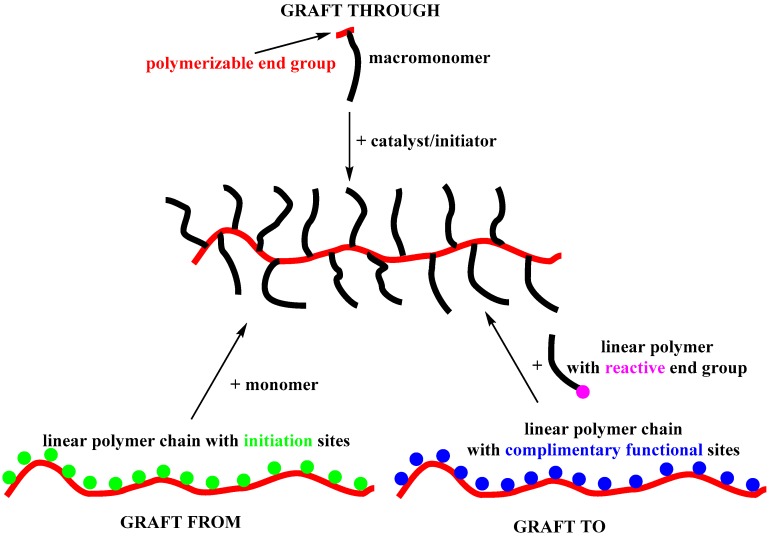
Methods for the synthesis of molecular brushes.

There are three main methods to prepare a densely grafted bottlebrush copolymer. Each grafting approach (from, to, through) exhibits certain advantages and disadvantages (Figure 6). There is no rule in employing only one of them, so a combination might be used, depending on the needs and synthetic limitations.

(a) “Grafting from”: The backbone of the bottlebrush copolymer bearing a number of initiation sites is synthesized first (macroinitiator). Then, the side chains are grafted from the macroinitiator by polymerizing a suitable monomer. The polymerization techniques used in both steps determine if the initiating groups can be incorporated directly or protected and introduced after the synthesis of the main chain. The backbone can be characterized fully, but the side chains need to be detached from the main chain for characterization (if possible). The conformation of the main chain renders the initiation sites not equally accessible. The main disadvantage of this approach is the probability of unreacted initiation sites. The molecular weight distribution of the side chains is another important issue, since broader distributions are expected due to slower initiation and propagation of sterically hindered sites. 

(b) “Grafting to”: The backbone and side chains are prepared separately and both can be fully characterized. The molecular brush is formed by reacting end-functional polymers with a polymer backbone precursor bearing complimentary functional groups on each monomeric unit. However, attaching a polymeric chain next to another polymeric chain might be difficult for steric reasons and the probability of unreacted sites on the main chain is not negligible. Also, slow diffusion of the bulky side chains towards the functional groups of the backbone increases the reaction time significantly. In both cases the side chain incorporation is incomplete and the copolymer is not a densely grafted one. Often a small excess of side chains is added to accomplish full incorporation in the main chain, so, another disadvantage, is the need to fractionate the bottlebrush copolymer from the remaining side chains. Thus, careful limitations of the main and side chain are in order for a successful synthesis of a bottlebrush copolymer and the ability to separate the product from the reactants.

(c) “Grafting through” (the macromonomer method): The macromonomer, that is polymeric chains bearing a polymerizable end group, are synthesized first and can be fully characterized. Polymerization of the end groups leads to the desired molecular brush architecture. The main advantage of this method is that each monomeric unit of the backbone bears a polymeric side chain without any doubt. The problems of low degree of polymerization and high polydispersities have been solved to a significant extent with **G3** (and **G3’**) catalysts, as far as ROMP is concerned. “Grafting through” is considered as the only method towards model molecular brushes.

In 2015, “transfer to” method was used in bottlebrush synthesis. This is a method employing Reversible Addition–Fragmentation chain Transfer (RAFT) agents attached to the backbone. The synthesis of the side chains and their attachment to the main chain is carried out simultaneously. It is a “grafting to” variation, where the side chains are not prepared and attached to the backbone separately, but in one single step. The results were not very encouraging and a mandatory purification step were the reasons why only two papers have been published using this method [39,40].

## 4. Catalysts/Initiators

A metal carbene, which is the catalyst, is necessary for metathesis reactions. The same metal center performs more than one metathesis reactions. In polymerization reactions, the term catalyst and (metal) initiator is sometimes confused. An initiator begins the polymerization reaction, and in living polymerizations remains bonded at the end of the polymeric chain until the termination of the reaction. Thus, an initiator molecule begins one polymeric chain, while a catalyst is responsible for the formation of more than one chain. The catalytic function of metal carbenes with a living character refers to the addition of monomers in the growing chain and not to the formation of many chains. If one metal center begins more than one chain randomly, the polymerization reaction cannot be controlled and large polydispersities are observed. This type of catalyst/initiator is not adequate for model polymer synthesis. In the text Grubbs’ and Schrock’s catalysts are mentioned as “catalysts” even though they act as initiators with a living character most of the times. This is for the reader’s convenience, since these complexes are globally known as such.

Synthesizing a backbone for “graft from” and “graft to” methods is quite easier than polymerizing macromonomers, because a small monomer is used. The difficult part utilizing “graft from” and “graft to” is growing the side chains and attaching the side chains to the backbone respectively. Scientists are interested in the “grafting through” method, because it is the only synthetic method where all the monomeric units of the backbone bear a polymeric side chain without any doubt! The polymerization of macromonomers is quite a difficult task, since a lot of factors have to be considered and problems not encountered in small monomer polymerization arise. The functional groups of the macromonomers, as well as the terminal functional group of each chain, might poison the metal complex. Thus, functional group tolerance is a major requirement of the metal carbene. Apart from that, well-controlled polymerization reactions with living character are required in order to prepare model molecular brushes or/and more complex bottlebrush architectures. Since the backbone bears carbon–carbon double bonds, the catalyst must not coordinate and react with those bonds, because undesired side products will be formed (network, chain transfer, etc.). The polymerizable end group needs to be accessible by the catalyst/initiator and long polymeric chains with random coil configurations render the polymerization of macromonomers harder and slower. So, an efficient synthesis demands fast initiators. Also, ill defined catalytic systems providing irreproducible results cannot be of any merit.

The backbone is synthesized by ROMP. Molybdenum and ruthenium complexes have been employed for this purpose (Figure 2). These complexes had already been used in the polymerization of small monomers. Yet, the synthesis of bottlebrushes by the “grating through” method needs different conditions than those for small monomers. The polymerization of an end group of a linear polymer is not as easy as the polymerization of small molecules for a number of reasons. The most important is the steric hindrance of the polymeric chain around the end group. Other factors, as the increased viscosity of the polymerization media and the macromonomer functional groups render the need for very active catalysts/initiators.

**Figure 7:**
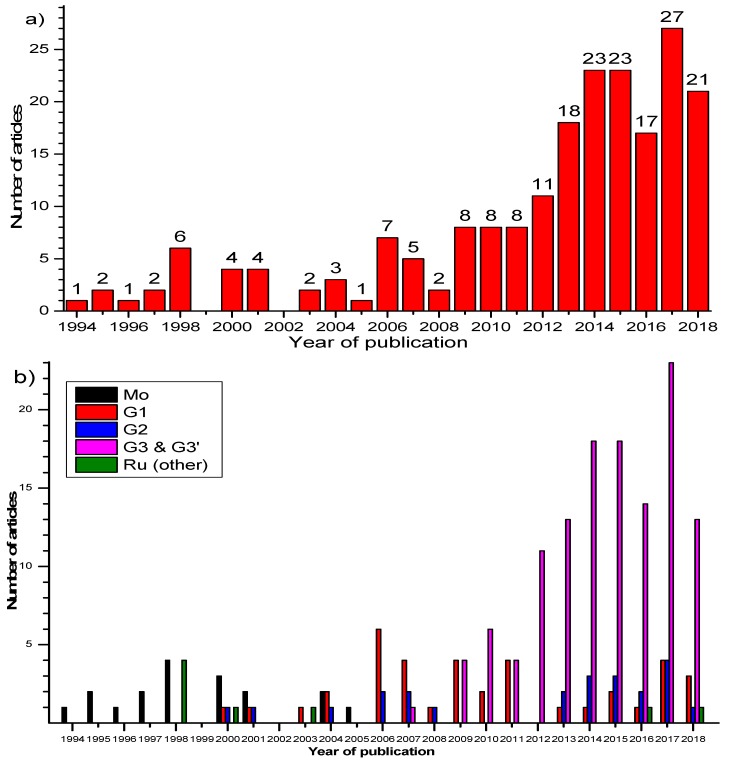
(**a**) Graphic representation of the number of publications concerning bottlebrushes per year until 2018; (**b**) Graphic representation of the number of articles published per year, where each catalyst is mentioned. (the articles where more than one catalysts were used, have been counted in each catalyst bar separately).

The well-characterized Schrock’s catalysts (Mo carbenes) were the first ones to be employed. The Mo carbenes are very sensitive towards the atmosphere and require special techniques for handling these materials. Unfortunately, the pioneering, for the time, work encountered many problems, which rendered the experimental procedures difficult. The results were not very satisfactory either, limiting the research interest [5,6,7,8,24,25,26,27,28,29,41,42,43]. The main disadvantages were the low tolerance towards functional groups of the (macro)monomers and the low degree of polymerization of macromonomers. Fourteen (14) synthetic papers have been published using Mo catalysts and the last one was in 2004 (Figure 7) [42].

Grubbs’ first- and second-generation catalysts were reluctantly employed in molecular brush synthesis for almost ten years since the first attempt in 2000 (28 publications) (Figure 7) [33]. This could be attributed to a number of reasons, like the reduced reactivity of **G1** compared to Schrock’s catalysts, easier methods to prepare molecular brushes, not good reaction control with **G2**, etc. **G2** was found to be more efficient in the polymerization of macromonomers bearing two side chains directly attached to the norbornene moiety.

The incorporation of Grubbs’ third generation catalyst (and the pyridine derivative) in the “grafting through” toolbox in 2009 [35,36,37,38] revealed great potential and more scientific teams directed their efforts towards the synthesis and applications of these macromolecular architectures. A number of factors contributed to this sudden change of interest. First of all, the catalyst is commercially available. Despite this fact, most researchers synthesize **G3’** from **G2** (also commercially available), because the reaction is quite easy experimentally. **G3** is air and thermally quite stable and exhibits great functional group tolerance compared to the other carbenes. High molecular weight polymacromonomers have been synthesized with a variety of side chains. Usually, the synthesized polymacromonomers have quite low molecular weight distributions, so model bottlebrushes can be studied. Short polymerization times (<1 h) are common and no byproducts are produced. The living character of the catalyst is exploited in more complex bottlebrushes, like (multi-)block brushes, stars with bottlebrush arms, modifications at the end of the main chain, etc. Summarizing, **G3** is commercially available or easy to prepare from **G2**. The polymerization is fast and well-controlled. Well-characterized (model) molecular brushes bearing a variety of functional groups can be synthesized. Put simply: ‘easy’, fast and good results. 

## 5. Syntheses of Molecular Brushes

**Figure 8:**
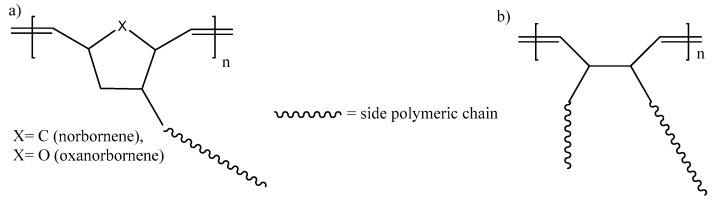
Structures of molecular brushes with (**a**) (oxa)norbornene- and (**b**) 1,4-butadiene- main chains.

**Figure 9:**
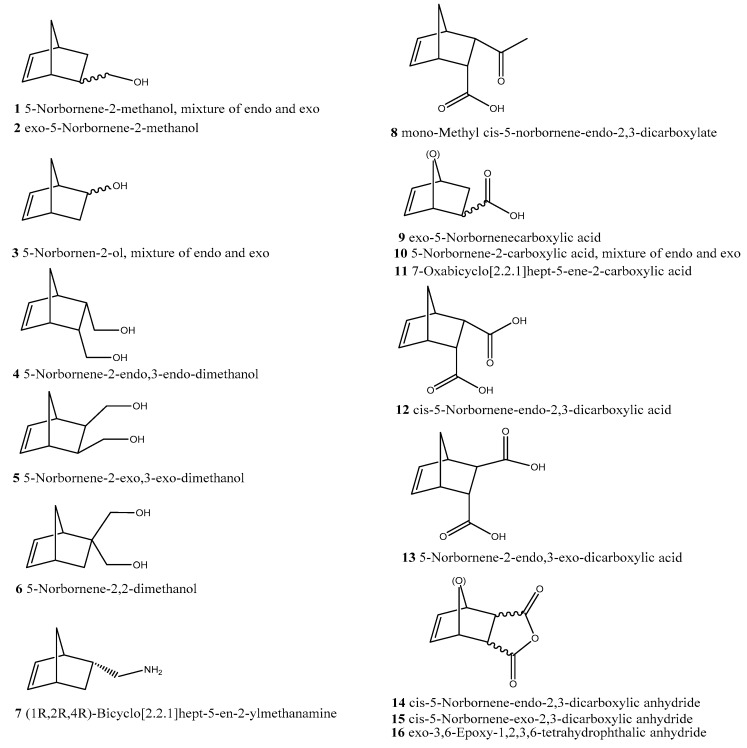
Most commonly used commercially available norbornene derivatives.

Even though more than three small monomers undergo ROMP, only three have been employed for the synthesis of the main chain of molecular brushes: (a) cyclobutene leading to 1,4-butadiene main chain (8 articles), (b) oxanorbornene (22 articles) and (c) norbornene (>160 articles) (Figure 8). The commercial availability of norbornene derivatives and the high polymerization rate of norbornene macromonomers are the main reasons for this fact (Figure 9).

### 5.1. Synthesis of Macromonomers

**Figure 10:**
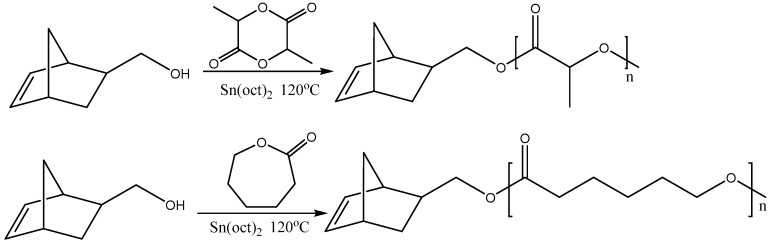
Examples of the syntheses of α-monotelechelic macromonomers of PLLA and PCL.

**Figure 11:**
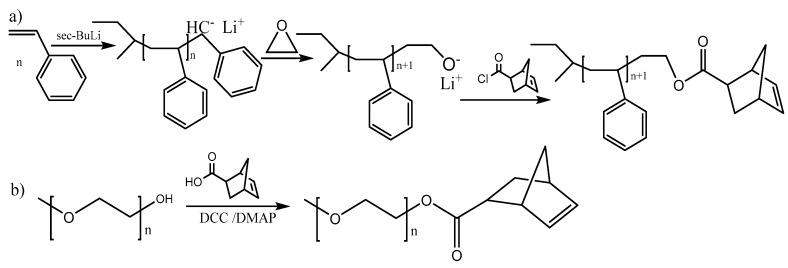
Examples of the syntheses of ω-monotelechelic macromonomers: (**a**) termination of anionic polymerization of styrene with NBE acyl chloride, (**b**) esterification of PEO–OH end group with NBE acid.

Usually, the preparation of the macromonomers requires much more time than the synthesis of the bottlebrush itself. There are two main routes to prepare macromonomers:

(a) α-monotelechelic macromonomers. The macromonomer is synthesized from an initiator which bears a norbornene (NBE) moiety (Figure 10). There are quite a few (oxa)norbornene derivatives commercially available (Figure 9), but they can also be synthesized by Diels–Alder reactions. Sometimes a small fragment (spacer) between the norbornene end group and the polymer is in order and norbornene anhydrides have been used for this purpose. Norbornene alcohols have been used as initiators for the ring opening polymerization (ROP) of lactides and lactones [44,45]. The amine could be used as initiator for the ROP of aminoacid N-carboxyanhydrides [46]. Employing organic chemistry reactions, initiators for any polymerization method of interest (RAFT, ATRP, etc.) have been synthesized.

(b) ω-monotelechelic macromonomers. There are two ways to synthesize these macromonomers: (i) termination of the polymerization with a suitable norbornene derivative, which attaches the norbornene moiety at the end of the polymeric chain, e.g. termination of anionic polymerization of styrene with an NBE acyl chloride [5], (ii) preparation of a polymer which bears a functional group which can react with a norbornene derivative, e.g., esterification of an alcohol end group with an NBE acid (or acyl) derivative [47] (Figure 11).

Depending on the side reactions the α- or the ω- route is chosen. The quality of the macromonomers is essential for the preparation of model molecular brushes [48]. If telechelic macromonomers (polymerizable group at both ends of the linear chain) exist in the monotelechelic macromonomer, then crosslinking of the molecular brushes leads to networks of high molecular weight or insoluble bybroducts. Side reactions (backbiting, chain transfer) during macromonomer synthesis, can also create linear polymers without NBE end groups. In this case, a significant amount of ‘unreacted macromonomer’ is observed in the Gel Permeation Chromatography (GPC) trace.

### 5.2. Endo-Exo Norbornene Isomers

It has been suggested that the endo–exo NBE isomers of the NBE end group react differently (different reaction rates) with the catalyst/initiator, due to steric hindrance, leading to incomplete consumption of the macromonomer [49]. Thus, some researchers select only the exo-isomer, which exhibits less steric hindrance towards the complexation of the NBE double bond to the metal center. It has been reported by our team that in experiments with polylactide and poly(hexyl isocyanate) macromonomers with endo:exo = 75:25 NBE end groups and **G1** catalyst, no significant amount of macromonomer was left unreacted (<1%) [50]. This small amount of “unreacted macromonomer” was attributed by researchers [34,50] to linear polymers not bearing NBE end units, rather than the steric hindrance of the endo macromonomers. The preparation of block molecular brushes with both macromonomers consisting of NBE endo:exo = 75:25 supports the latter hypothesis (~1% “macromonomer” did not react in the formation of the first block even after days of reaction and the addition of new amount of catalyst after those days).

## 6. Side Chains of Molecular Brushes and Applications

### 6.1. Poly(ethylene oxide) Side Chains

Poly(ethylene oxide) (PEO) or poly(ethylene glycol) (PEG) exhibits a variety of properties which have been exploited in medical, biological, chemical, industrial and commercial applications. Its synthesis from ethylene oxide (EO) by anionic polymerization is not an easy task, since EO is a toxic gas, and most researchers prefer the commercially available polymers. The most common commercially available PEO possesses a hydroxyl end group when synthesized. Modified PEO with other than OH functional end groups and star architectures are also commercially available. Thus, ω-monotelechelic NBE macromonomers are usually prepared employing organic and catalytic reactions. A total of 59 articles where PEO bottlebrush segments are involved have been published.

PEO molecular brushes have been synthesized by “graft through” and “graft to” methods. The initial “graft through” bottlebrush PEO synthetic attempts were fulfilled with Mo carbenes in 1996–1998 [7,8,27]. Until 2010 no other attempt was made to synthesize PEO bottlebrushes. Then, **G3** was employed to polymerize PEO macromonomers (*M*_n_ ~ 3000) with very good control (1.04 < *I* < 1.27) for DPs up to 400 [51]. After 2015, there are a few papers where **G1** and **G2** have been used to polymerize PEO macromonomers [13,52,53,54] and a comparison between **G1**, **G2, G3** and **G3’** was made by some researchers [53,54]. In general, **G3** (and **G3’**) provided the best results and the seeding technique [54] was proven useful. It has been observed that increasing the molecular weight of the macromonomer leads to decreased control of the synthesis of the molecular brush. High ratio PEO:catalyst (>50) can lead even to unsuccessful reaction [55]. The maximum molecular weight of PEO macromonomers mentioned in the literature is 5000. In most cases and especially with shorter macromonomers the polymerization was well-controlled and a living character of the polymerization could be observed and exploited in the formation of more complex architectures. Obviously, Grubbs’ catalysts have solved several problems, but it seems that polymerizing PEO macromonomers of high MW or synthesizing PEO polymacromonomers with high DP is still a challenge.

PEO molecular brushes have been incorporated in more complex architectures (Figure 4) due to the living character of Grubbs’ catalysts. Copolymers with polylactides [56,57,58], polystyrene and polystyrene derivatives [8,27,47,57,58,59,60,61,62,63,64,65,66,67,68,69], ε-caprolactone [52,53], (meth)acrylates [70,71,72,73], peptides [35,74,75,76,77] have been synthesized and studied. The covalent attachment of drug molecules on the PEO side chains or as a separate block on the main chain has also been employed for drug delivery purposes [78,79,80,81,82,83,84]. Other small molecules serving a specific function have also been covalently bonded on the PEO side chains or in umbrella-like structures [72,73,74,85,86,87,88,89,90,91,92,93,94,95,96]. In some cases, more complex structures have been synthesized, depending on the needs of applications, or for academic interest.

Block molecular brushes (Figure 4A) that have been reported in the literature are: (a) diblocks PS-*b*-PEO [8,27,62,63,64,65,66], PEO-*b*-P*t*BOS [68], PEO-*b*-(poly)peptide [75,76,77], P*t*BA-*b*-PEO [70,71], and (b) triblocks PEO-*b*-PDLLA-*b*-PBAEAM [57], PS-*b*-PEO-*b*-PS [61]. Molecular brushes with random side chains (Figure 4B) have been synthesized: PS-*co*-PEO [27,59], PEO-*co*-PCL [52], PE-*co*-PCL-*co*-PEO and PE-*co*-PCL-*co*-PEO-*co*-PS [47], P*t*BOS-*co*-PEO [68] and random copolymers of PEO with three different end groups [97]. Bottlebrushes with two PEO chains per monomeric unit (Figure 4C) have been prepared [52]. Pseudo-alternating bottlebrushes with two different chains per monomeric unit (Figure 4D) are: PEO/PCL [53], PEO/PLLA [56], PEO/nitroxide marker [85,86,89] and PEO/PS [98]. Pseudo-alternating structures with one PEO polymeric chain and a small molecule (drug or others) on each monomeric unit have been synthesized for high (bio)technology materials: PEO-doxorubicin [78,84], PEO-camptothecin [78], PEO-triethylene glycol [99]. Molecular brushes with umbrella-like structure (Figure 4F) have been prepared with a variety of norbornene derivatives for specialized purposes [79,83,87,88,90,91,92,93,94,100]. Core–shell structures (Figure 4E) have been synthesized with PS-*b*-PEO, PEO-*b*-PS [27] diblock side chains. The living character of Grubbs’ catalysts provided the means to synthesize complex architectures consisting of a block of PEO side chains, a block of poly(2-ethylhexyl methacrylate) side chains and two more blocks of small molecules attached to an NBE main chain [72,73,74]. Stars bearing brush PEO arms with covalently bonded anticancer drugs have been synthesized by crosslinking the arms with stimuli-degradable crosslinkers [58,80,81,82]. Similar structures without drugs have been synthesized with PEO (and/or PS) brush arms recently [67,101]. A brush on brush advanced structure, where polymeric chains have been grown from initiation sites on the bottlebrush side chains, has been reported in 2018 [68].

PEO is involved in drug delivery systems due to its “stealth” property. It is not recognized by the immune system and the defense mechanism of the organism is not triggered. Thus, drug carriers bearing PEO moieties on their outer surface can move undisturbed inside the organism towards the target tissue. When the carriers reach the (cancer) tissue, they are disassembled and the drug is released. This is a simplistic description. In vivo, drug delivery encounters many problems in its application and different strategies have been employed for better solutions.

While studying under R.H. Grubbs, J. Johnson had synthesized PEO molecular brushes with covalently bonded doxorubicin [78,84] or camptothecin [78]. Photodegradable linker molecules were used to attach the drug onto the macromolecules, which were later exploited in a controlled release of the anticancer agent in response to 365 nm UV light. The polymers containing doxorubicin or camptothecin were at least 10 times more toxic to human cancer cells after photoinitiated drug release while a copolymer carrying both the drugs displayed 30 times increased toxicity upon irradiation [78]. Continuing this trend, but with his own team, J. Johnson synthesized and tested more complex brush-arm star polymer (BASP) macromolecular architectures. These structures were synthesized by crosslinking PEO molecular brushes bearing doxorubicin with photo or acid degradable crosslinkers [80,81]. In order to improve the therapeutic effects three drugs doxorubicin, camptothecin and cis-platin have been incorporated in the same BASP. Drug release occurs in response to three distinct triggers. The ratio of each drug can be tuned precisely by the ratio of the corresponding macromonomer. This concept for the combination of drugs was made possible using highly convergent synthesis of nanoparticles [82].

Qiao and coworkers have synthesized cross-linked polymer vesicles using thiol–ene chemistry. The umbrella-like structures were allowed to self-assemble and then the double bonds of the main chain were used to crosslink the formed structures. Cis-platin was conjugated in these structures at a high loading content and it was released at a steady rate [87]. The same team has published another article, where an acid degradable crosslinker was used in order to improve targeted delivery. The cross-linked drug carriers degraded at lysosomal acid condition (pH ~ 5.5). The activity of cis-platin loaded into the cross-linked vesicle was improved in comparison to the free drug [88].

Kasi and her team have prepared nanoparticles by self-assembling amphiphilic umbrella-like PEO brushes containing cholesterol side groups. Doxorubicin was loaded in these nanoparticles at high loading content and in vivo circulation time and tissue distribution was studied in mice. The nanoparticles had greater tumor accumulation with significantly reduced toxicity compared to the free drug in a mouse xenograft tumor model [79,96].

Another approach was realized by J. Cheng and coworkers, by synthesizing diblock molecular brushes with PEO and polypeptide side chains. Polyglutamate and polylysine were chosen as the polypeptide segments. Paclitaxel was loaded in the formed nanoparticles and their stability was studied [75]. C. Cheng and his team have also incorporated paclitaxel in their drug delivery study. They prepared umbrella-like (referred to as “diblock” in their paper) and random copolymers using NBE functionalized paclitaxel with cycloacetal based cleavable conjugation linkages and PEO macromonomers. The influence of each structure towards the drug release and the therapeutic properties was studied and the results were encouraging [83]. Hobbs, Zhu and their teams have synthesized micelles from umbrella-like PEO structures. They coloaded paclitaxel and curcumin in these carriers and a synergistic anticancer effect was exhibited in both the drug mixture and drug coloaded micelles at high curcumin/paclitaxel ratio [91]. 

Ohe and his team have utilized complex molecular brush structures containing PEO for tumor targeting and imaging. The living character of ROMP was crucial for the incorporation of the targeting and imaging components on the same NBE backbone along with the PEO bottlebrush segment. In vivo imaging studies were conducted in cancer infected mice. Their results offered great potential for noninvasive and effective optical imaging, but also for drug delivery applications [72,73,74,90]. Zhang and his coworkers have exhibited the potential of molecular brushes of oligonucleotides in molecular imaging and targeted therapy through an in vivo study with mice [100]. Molecular brush structures containing cell-penetrating oligopeptides have been synthesized and studied by Gianneschi and his team. Their study presented a simple, effective and broadly applicable alternative strategy that enabled cell penetration of peptides for therapeutic or diagnostic purposes [76]. Another application in medical problems was realized in 2018 by Kasi, Wei and their coworkers. The mineralization of collagen fibrils induced by molecular brushes was studied and the results were better than those provided by linear polymers [97].

Apart from the aforementioned medical-biological applications, bottlebrushes with PEO segments have been synthesized and studied as potential materials for various applications. Fan and his team have studied the potential of molecular brushes to be used as high-temperature lithium ion batteries [92]. On the same track, Grubbs, Jones and their coworkers have investigated the ionic conductivity of PS-*b*-PEO-*b*-PS triblock molecular brushes doped with lithium bis(trifluoromethane)-sulfonimide [61]. Kasi and her team have prepared and studied umbrella-like structures containing liquid crystalline (cholesterol) molecules as side groups on the backbone with potential use in stimuli responsive applications [94,95]. Watkins and his team were interested in synthesizing and investigating the ability of bottlebrushes to be incorporated in photonic crystal materials. Much higher loadings of functionalities and greatly expanded domain spacings were characteristic of the hybrid materials. Quantum dots have also been assembled in well-ordered photonic nanocomposites [62,63,64,65,70,71].

### 6.2. Polystyrene (and Polystyrene Derivative) Side Chains

Polystyrene (PS) is one of the common materials in many commercial applications. A variety of complex macromolecular architectures has been synthesized and molecular brush structures with PS side chains have been reported in the literature. In this section, bottlebrushes with side chains of PS and PS derivatives will be discussed (61 articles).

PS was the favorite macromonomer for the initial attempts with Mo catalysts (1994–2000) [4,5,6,8,24,25,26,27,43]. In 2003, Liaw and his coworkers published a paper reporting that PS macromonomers could not be polymerized with **G1 [102]**. In 2004, Khosravi and his team polymerized PS macromonomers bearing one or two chains per NBE unit with **G1 [48]**. Khosravi encountered problems, like unreacted macromonomer and medium yields, but managed to prepare molecular brushes. The main difference of these two attempts was the synthetic procedure for the macromonomers. Liaw prepared PS macromonomers employing ATRP and Khosravi prepared PS macromonomers using anionic polymerization and termination with NBE–COCl. Since the matter was not analyzed by Liaw, only assumptions can be made for this contradiction. Despite Khosravi’s partial success, bottlebrushes synthesized with **G1**, containing PS side chains were prepared exclusively with the ”graft from” method (ATRP [10,103,104,105,106,107,108], RAFT [109,110]) until the establishment of **G3** and **G3’** in 2009. The last paper where “graft from” was used for PS was published in 2011 [108]. PS macromonomers have been employed by other teams (Hadjichristidis, Pitsikalis) in copolymer synthesis with **G1** and no significant problems were mentioned [47,68]. Ultra-high molecular weight PS bottlebrushes have been synthesized using **G3’ [69]**.

Block molecular brushes (Figure 4A) that have been reported in the literature are: (a) diblocks PS-*b*-PEO [8,27,62,63,64,65,66], PEO-*b*-P*t*BOS [68], PS-*b*-P*t*BOS [68,69], PS-*b*-PBd [26,27,43], PS-*b*-PLLA [104], PS-*b*-PDLLA [111], PS-*b*-P*t*BA [112], PTF*p*HS-*b*-(P*p*HS/PhMI) [113,114], P(fluoro methacrylates)-*b*-(P*p*HS/PhMI) [114,115] and (b) triblocks PS-*b*-PEO-PS [61]. Molecular brushes with random side chains (Figure 4B) have been synthesized: PS-*co*-PEO [27,59], PE-*co*-PCL-*co*-PEO-*co*-PS [47], P*t*BOS-*co*-PEO, P*t*BOS-*co*-PS, PSOH-*b*-PS [68]. Bottlebrushes with two PS chains per monomeric unit (Figure 4C) have been prepared [4,10,24,44,104]. Pseudo-alternating bottlebrushes with two different chains per monomeric unit (Figure 4D) are: PS/PEO [98], PS/PLLA [104,116], PS/PDLLA [58,99], PS/P2VP [117], PS/PDMS [118,119], PS/P*t*BA [118]. Molecular brushes with umbrella-like structure (Figure 4F) have been prepared with a variety of norbornene derivatives for specialized purposes [103,105,106,108]. Core–shell structures (Figure 4E) have been synthesized with PS-*b*-PEO, PEO-*b*-PS [27], PS-*b*-P4VP [108], (PS-*co*-PMAn)-*b*-PS and PS-*b*-(PS-*co*-PMAn)-*b*-PS [109,110], PE-*b*-PS [120]. Brushes with random copolymers as side chains have been synthesized: (PS-*co*-PMAn)-*b*-PS, PS-*b*-(PS-*co*-PMAn)-*b*-PS [109,110], PS-*co*-PMMA [121]. PSOH polymacromonomers were employed as initiators for EO polymerization towards brush on brush structures [68].

The importance of impurities in PS macromonomers was demonstrated by Xia and Teo. The quality of the macromonomers is essential for the synthesis of molecular brushes without crosslinked byproducts and/or significant amounts of “unreacted” macromonomer [48]. The termination or side reactions during the macromonomer synthesis have to be eliminated, or at least minimized as much as possible. Similar observations in the ROP of lactides and caprolactone, where transesterification or back-biting reactions led to similar byproducts have been made by our team (unpublished results). Thus, the synthesis of the macromonomers is no mean feat and requires attention, since it is generally difficult to assess the quality of the macromonomers by the polydispersity index alone (GPC). 

The significance of the norbornene fragment (anchor group) of the macromonomers and the relative reaction rate was investigated by Matson and his colleagues [122]. Three of the most common anchor groups have been employed and their effect on the kinetics of the polymerization of the macromonomers with **G3’** were studied. Experimental and computational studies showed that the differences more likely derive from a combination of steric effects and the electronic structure of each monomer. Thus, the choice of the anchor group is a critical factor when designing synthetic strategies for preparing molecular brushes.

The initial investigations of the conformations of PS bottlebrushes in solution were performed by viscosimetric and light scattering experiments [123,124,125]. The molecular brushes adopted spherical, cylindrical, or wormlike morphologies depending on the molecular weight and DP. Their dynamics was described by a single relaxation time. Dense and not entangled particles were revealed by dynamic light scattering (DLS) and viscosimetry [125]. Grubbs and his colleagues investigated the morphology of PS bottlebrushes in the solid state and found cylindrical shapes and suggested that the molecular brushes had extended backbone conformation with the side chains stretched and flattened on the surface [37].

The capability of synthesizing high molecular weight bottlebrushes and their self-assembly property interested scientists who study and exploit size and morphology-dependent physical properties. Bowden and his team investigated the morphology of PS bottlebrushes with various architectures in the solid state (Figure 4). Different molecular weights and compositions were studied. They found that rigid rods with lengths exceeding 300 nm [106] and spherical, lamellar, and cylindrical arrays exceeding 100 nm could be formed [104,105,107]. The authors mention the potential applications of the optical properties of these morphologies, especially as photonic materials. Watkins and his colleagues were interested in optical applications of bottlebrushes and synthesized thermally tunable metallodielectric 1D photonic crystals with high loading of metal (Au) [62]. The reflection of light was widely tunable from the visible to near infrared region. Significant effects on the photonic characteristics were provided by the control over size as well as the distribution of the gold nanoparticles in the well-ordered structure through simple thermal treatment. The same team, managed to direct the assembly of quantum dots within photonic nanocomposites. Strong photoluminescence and third harmonic generation were observed via multiphoton excitation using femtosecond laser light at several wavelengths from 700 to 1550 nm [64]. Liaw and his team synthesized carbazole-containing umbrella-like structures, observed strong emissions of these fluorescent copolymers and suggested a potential use as hole transport materials in molecular electronic devices [103]. Grubbs and his colleagues synthesized PS-b-PDLLA diblock brush, which self-assembled in ordered lamellar nanostructures with photonic bandgaps spanning the entire visible spectrum, from ultraviolet to near infrared. They envisioned a potential application as near infrared reflecting building materials to inhibit the thermalization of urban environments [111]. Fundamental dielectric mirrors were assembled from PS-*b*-P*t*BOS diblock brush as well [69].

Fluoro-containing bottlebrushes have been studied as photolithographic materials by Wooley and her team. The chemically amplified negative-tone materials showed higher resolution and higher sensitivity than a linear block copolymer. The thinner cylindrical diblock molecular brushes could generate narrower line widths, whereas the thicker ones allowed electron-beam lithography without a post exposure-baking step. The bottom up synthetic strategy allowed fine-tuning of the full dimensions to balance the properties and performance during top-down lithographic processing [113]. On the same concept, block molecular brushes including poly(fluoro methacrylate)-based blocks that function as substrate vertical alignment promoters were synthesized [114]. An attractive material for nanolithography was synthesized by Ross and her colleagues. The Janus type pseudo-alternating PS/PDMS (Figure 4D) bottlebrush copolymer exhibited 22 nm period cylindrical microdomains with long-range order under solvent vapor annealing. The PS matrix can be removed when exposed to oxygen plasma; leaving behind oxidized PDMS microdomains that serve as robust etch masks [119].

Verduzco, Stein and their teams investigated the synthesis and application of stimuli-responsive molecular brushes. The only copolymer with mixed polynorbornene-polyoxanorbornene main chain (PS-*co*-PEO) was investigated as surface coating stimuli-responsive material [59]. In 2018, a PS polymacromonomer bearing –SH end-functionalized side chains was studied as a potential surface coating [126]. The application of bottlebrushes as additives has also been studied and their impact on the phase separation of polymer blends was exhibited [121,127].

The synthesis, self-assembly, conductivity, and mechanical properties of densely grafted PS-b-PEO-b-PS triblock brush terpolymers were reported by Grubbs and his colleagues. The lithium doped material could find application in batteries, which are extensively used in many modern devices (computers, cell phones, automobiles, etc.) [61]. PS/PEO molecular brushes have also been used as macromolecular reactive surfactants in the synthesis of well-defined polymeric latex nanoparticles [98].

The morphology of bottlebrushes containing PS side chains has been studied extensively, since many applications require special features in the solid state. Most of the articles mentioned deal with the morphology of the synthesized bottlebrushes and they shall not be mentioned separately.

### 6.3. Polylactide Side Chains

Lactide is the cyclic diester of lactic acid. The ring opening polymerization reaction of lactide leads to polylactide (PLA), which is referred to as poly(lactic acid) sometimes. There are four commercial products available: (a) *l*-lactide, LLA, (CAS: 4511-42-6), (b) *d*-lactide, DLA, (CAS: 13076-17-0), (c) the racemic mixture of *d*- and *l*-lactide, DLLA, (CAS: 95-96-5) and *meso*-lactide (CAS: 13076-19-2). The majority of the studies on lactide bottlebrushes involve PDLLA side chains (23 articles) [36,49,58,99,111,122,123,124,125,126,127,128,129,130,131,132,133,134,135,136,137,138,139,140,141,142,143,144]. There are only 10 articles with PLLA side chains [12,34,44,50,56,57,104,108,116,145,146,147,148] and one article where PDLA (and PLLA) side chains are used [145].

Lactides are monomers deriving from renewable resources by fermentation and are considered green monomers. Polylactides have been used in many commercial and special applications (3D printers, disposable tableware, packaging, etc.). Their biocompatibility and biodegradability renders them very useful biomaterials (sutures, medical implants, etc.).

In 2004, Bowden and his colleagues were the pioneers to synthesize ultralarge molecular weight PLLA bottlebrush polymers using **G1** and **G2** with one and two arms per monomeric unit (Figure 4) [34]. Despite their undeniable success, and the general interest and applicability of PLAs, only 9 more articles involving molecular brushes with PLA side chains appeared till 2014. In the last five years (2014-2018), 26 articles were published showing a growing interest in PLA bottlebrushes.

PDLLA molecular brushes have been synthesized by “graft through”, while PLLA bottlebrushes have been prepared by “graft through” and “graft from” methods. Only Grubbs’ catalysts have been employed for ROMP of PLA macromonomers.

Block molecular brushes (Figure 4A) that have been reported in the literature are: (a) diblocks PS-*b*-PLLA [104], PS-*b*-PDLLA [111], PDLLA-*b*-P*n*BA [36], P3HT-*b*-PDLLA [143], PLLA-*b*-PHIC [50], PLLA-*b*-PCL [44] and (b) triblocks PLLA-*b*-PHIC-*b*-PLLA [50], PHIC-*b*-PLLA-*b*-PHIC [50], PEO-*b*-PDLLA-*b*-PBAEAM [57] and dumbbell shaped PDLLA_45_-*b*-PDLLA_15_-*b*-PDLLA_45_ with PDLLA side chains of different MW [49]. Molecular brushes with random side chains (Figure 4B) have been synthesized: PLLA-*co*-PHIC [50], PLLA-*co*-PCL [44], PDLLA-*co*-P*n*BA [36], PDLLA-*co*-PDMS [138], P3HT-c*o*-PDLLA [139,143]. Bottlebrushes with two PLA chains per monomeric unit (Figure 4C) have been prepared [12,34,44,56,144]. Pseudo-alternating bottlebrushes with two different chains per monomeric unit (Figure 4D) are: PEO/PLLA [56], PS/PLLA [104,116], PS/PDLLA [58,99]. Molecular brushes with umbrella-like structure (Figure 4F) have been prepared with a variety of norbornene derivatives for specialized purposes [108,132,133,134,135,140,141,144]. Core–shell structures (Figure 4E) have been synthesized with PLLA-b-PHIC diblock side chains [148]. Core-photodegradable miktoarm star polymers bearing brush PDLLA/PS arms have been synthesized by crosslinking the arms with photo-degradable crosslinkers [58]. 

The biocompatibility of polylactide was exploited in studies involving indomethacin (analgesic, anti-inflammatory and antipyretic) with molecular brush carriers. There was no cytotoxicity detected towards human embryonic kidney cells [144]. A PS/PDLLA molecular brush with potential application in combination drug delivery and molecular imaging has also been prepared recently [99].

Kasi, Osuji and their teams have prepared and studied umbrella-like structures with PLA side chains. These liquid crystalline materials have shown magnetic field directed self-assembly [132,133] and membranes with aligned pores have been prepared [141]. The morphology of the bottlebrushes has been studied and well-ordered mesophases were observed with transitions from spheres to hexagonally packed cylinders, lamellae, inverse cylinders, and inverse spheres depending on the weight fraction of the liquid crystalline block [134]. The authors were interested in generating aligned nanoporous materials using low-cost permanent magnets [140]. Ahn, Lee and their coworkers synthesized random and diblock P3HT-PLA bottlebrushes, which self-assembled in different structures in a selective solvent that are differentiated based on their side chain arrangement as well as copolymer composition. Nonpersistent nanofibrils, highly persistent nanofibrils and spheres were observed depending on the topology of the bottlebrushes [143]. Their potential application as templates for optoelectronic applications or membranes for separations is mentioned by the authors [139]. The self-assembly of PS-*b*-PDLLA block brush copolymer was exploited by Grubbs and his team, who prepared photonic crystals with tailored bandgaps [111]. An interesting idea was realized exploiting the living character of Grubbs’ catalysts. A dumbbell shaped molecular brush was synthesized by sequential polymerization of macromonomers of different molecular weight. The actual architectures were visualized by atomic force microscopy [49]. A brush block copolymer of PDLLA-*b*-P*n*BA was shown to self-assemble into highly ordered lamellae with domain spacing >100 nm [36]. PDLLA containing bottlebrushes were synthesized and tested as surface active additives by Verduzco and his team. Chemically identical linear polymers were the main material and the spontaneous accumulation of the bottlebrushes at surfaces through an entropy-mediated process was exploited [138].

The morphology of PLLA bottlebrushes was initially studied by Bowden and his team. Spheres or rigid rods were detected by light scattering experiments [34]. Janus nanomaterials were self-assembled by thermal annealing of PLLA/PS pseudo-alternating bottlebrushes. The “gradual Janus” and left−right Janus conformations were detected by transmission electron microscopy [116]. Materials for applications in photonics and the synthesis of porous three-dimensional arrays were synthesized by Bowden and his team. The molecular brushes had arrays with domain sizes exceeding 100 nm [104]. In 2014, Fontaine and his team proposed that the hydrolytic degradation potential of the polyester side chains of their bottlebrush copolymers could be exploited in the preparation of complex hollowed nanostructures [12]. Micelles were formed from triblock brush copolymers in nanopure water by Wooley and her colleagues. Their self-assembly behavior was investigated before and after functional group transformation and different morphologies with interesting architectural features were revealed [57]. Cheng and his team prepared amphiphilic brush copolymers and their performance as emulsion surfactants was investigated. These giant macromolecular surfactants resulted in miniemulsions with remarkably enhanced stability [56]. A series of molecular brushes containing PLLA and PDLA side chains were synthesized by Satoh and his team. Stereocomplex formation and structure−property relationships were studied. The thermal properties (melting temperature, crystallinity) of the resulting stereocomplex varied depending on the backbone length, relative chain direction, and distribution of the PLLA/PDLA sidechains [145]. The thermal properties and thermal decomposition of PLLA containing molecular brushes was studied by Pitsikalis and his team [44,50,148]. The physics of semicrystalline polymers of complex topologies was studied in great detail [44]. Bottlebrushes with helical chiral poly(hexyl isocyanate) chains induced by a PLLA block were studied and their transformation to random coil with increasing temperature was observed [148]. A unique observation of close proximity of PLLA and PHIC side chains was made by 2D NOESY NMR in a diblock brush copolymer [148]. This was originally suggested by the minute increase in the molecular weight of the bottlebrush when the PHIC block was added.

### 6.4. Poly(ε-caprolactone) Side Chains

*ε*-caprolactone is the cyclic ester of 6-hydroxyhexanoic acid (6-hydroxycaproic acid). As with lactides, the ring opening polymerization reaction of this cyclic monomer leads to poly(*ε*-caprolactone) (PCL), which is a semi-crystalline, hydrophobic polymer with a melting point of 59–64 °C and a glass-transition temperature of −60 °C. Apart from other applications, the biocompatibility and biodegradability attracted great attention for use as implantable biomaterials. Molecular brushes bearing PCL side chains have been mentioned in 16 articles. 

The first attempts to incorporate PCL as side chains in molecular brushes was made in 1998 by Jerome and his team, using [RuCl_2_(*p*-cymene)]_2_/PCy_3_/(trimethylsilyl)diazomethane as the catalytic system for “graft from” and “graft through” ROMPs [31,32]. Despite their success, ten years passed (2008) until Xie and his colleagues polymerized PCL macromonomers again (**G2**). Xie’s bottlebrushes exhibited narrow polydispersity (1.2) for DP = 5, but larger ones (1.7–1.8) for DP > 25 [149]. 

Diblock molecular brushes (Figure 4A) that have been reported in the literature are PLLA-*b*-PCL [44] and PE-*b*-PCL [47]. Molecular brushes with random side chains (Figure 4B) PLLA-*co*-PCL [44], PEO-*co*-PCL [52], PE-*co*-PCL, PE-*co*-PCL-*co*-PEO, PE-*co*-PCl-*co*-PEO-*co*-PS [47] have been synthesized. Bottlebrushes with two PLA chains per monomeric unit (Figure 4C) have been prepared [12,44,150]. Pseudo-alternating bottlebrushes with two different chains per monomeric unit (Figure 4D) are PCL/PDMAEMA [149] and PEO/PCL [53]. Molecular brushes with umbrella-like structure (Figure 4F) and pseudo-alternating PCL/PEO side chains have been prepared [53]. Core–shell structures (Figure 4E) have been synthesized with PE-b-PCL diblock side chains [47]. Bottlebrushes with P(CL-*co*-ClCL) side chains have also been synthesized and Cl was used for side chain functionalization [151]. 

In 2012, Qiao and his team synthesized a cylindrical bottlebrush polypseudorotaxane with PCL and α-cyclodextrins [152]. This inclusion complex structure was thoroughly characterized and Transmission Electron Microscopy (TEM) analysis revealed their elongated cylindrical morphology. Potential applications in controlled drug and gene delivery, and tissue engineering scaffold were envisioned by the authors. Kasi and her team studied the shape memory properties of crosslinked molecular brushes bearing PCL side chains intended for biomedical and tissue engineering applications [45]. Leroux and his colleagues studied the self-assembly of PCL bottlebrushes in lamellae with large scale d-spacings. Their potential as building blocks for nanomaterials is mentioned [153]. The morphology and thermal properties of PCL/PLLA statistical and block molecular brushes with one and two chains per monomeric unit have been studied in detail by Pitsikalis, Floudas and their colleagues [44]. 

### 6.5. Poly(acrylate) and Poly(methacrylate) Side Chains

Poly(acrylate) and poly(methacrylate) linear polymers have found significant applications in the modern society. Molecular brushes bearing such side chains have been synthesized and their properties studied by several scientific teams. Poly(methyl methacrylate) (PMMA), poly(t-butyl acrylate) (P*t*BA), poly(n-butyl acrylate) (P*n*BA) and poly(2-(dimethylamino)ethyl methacrylate) (PDMAEMA) are some of the most common side chains, but bottlebrushes for special applications demanding exquisite side chains have also been prepared. A total of 47 papers involving poly(acrylate) and poly(methacrylate) side chains has been published.

The first successful bottlebrush bearing PMMA side chains was prepared in 2003 by Li and his coworkers. A polymer supported Ru complex was employed for this synthesis, but large polydispersities (>2.50) were achieved for the bottlebrushes [154]. In 2006, Fontaine and his team polymerized PMMA cyclobutenyl macromonomers and the products exhibited low polydispersities (<1.27) [9]. “Grafting through” PMMA norbornene macromonomers towards bottlebrushes with low polydispersities was achieved in 2012 using **G3’ [155]**. 

Molecular brushes have been synthesized by “graft through” and “graft from” methods. All generations of Grubbs’ catalysts have been used with a variety of macromonomers. Diblock molecular brushes (Figure 4A) that have been reported in the literature are PI-*b*-P*t*BA [156,157], PI-*b*-PAA [156], PDLLA-*b*-P*n*BA [36], PS-*b*-P*t*BA [112], PTF*p*HS-*b*-(P*p*HS/PhMI) [114], P(fluoro methacrylates)-*b*-(P*p*HS/PhMI) [114,115], PEO-*b*-P*t*BA [70,71,97], PEO-*b*-PAA [97]. Molecular brushes with random side chains (Figure 4B) PDLLA-*b*-P*n*BA [36] have been prepared. Umbrella-like molecular brushes (Figure 4F) have been prepared with PMMA [103] and P*n*BA [108] side chains. Bottlebrushes with two P*t*BA [9,10,158], PMMA [10] and PDMAEMA [159] chains per monomeric unit (Figure 4C) have been prepared. Pseudo-alternating bottlebrushes with two different chains per monomeric unit (Figure 4D) are PCL/PDMAEMA [149], P*t*BA/PDMS [118], P*t*BA/PS [118] have been synthesized. Brushes with random PS-*co*-PMMA as side chains have been synthesized [121]. Core–shell structures (Figure 4E) have been synthesized too [156]. More complex architectures consisting of a block of PEO side chains, a block of poly(2-ethylhexyl methacrylate) side chains and two more blocks of small molecules attached to an NBE main chain have been prepared [72,73].

The morphology of the bottlebrushes has been studied by many researchers. Wooley and her team observed PMMA ellipsoidal shapes with variable sizes by Atomic Force Microscopy (AFM) measurements. Aggregation of the polymer gave interesting surface nanosized irregular patterns [160]. Core-cross-linked PI-*b*-P*t*BA nanoparticles exhibited rigidified cylindrical shape and narrowly-dispersed size as revealed by AFM [157]. Fontaine and his colleagues observed individual wormlike molecules of P*t*BA bottlebrushes with two side chains per monomeric unit by AFM [10]. Grubbs and his colleagues observed highly ordered lamellae with domain spacing over 100 nm of PDLLA-*b*-P*n*BA brush block copolymers by SAXS. AFM also revealed large cylindrical micellar structures of 200-nm wide and several mms long [36]. Watkins and his colleagues observed highly ordered lamellae of P*t*BA-*b*-PEO brush block copolymer. They reported an exceptionally large volume of highly ordered arrays on the order of millimeters, which is 10^9^ times larger than that of typical self-assembled linear block copolymers [70]. A detailed study of the same bottlebrushes, P*t*BA-*b*-PEO, with varying side chains lengths was conducted by the same team [71]. Janus bottlebrush block copolymers have been assembled from pseudo-alternating PS/P*t*BA and PDMS/P*t*BA molecular brushes and studied by Johnson and his team [118]. Qiao and his team have synthesized PMMA molecular brushes with stereoregular (syndiotactic) side chains and an astonishing organic nanocrystal stereocomplex through triple-helix formation was assembled [161]. PMMA-Fullerene peapod nanoparticles have also been assembled by the same team [162]. Hong, Xu and their colleagues studied the structure–property relationships of PTFEMA bottlebrushes by TEM and AFM imaging. Their results provide significant information for tailoring materials with desirable properties [163]. Direct observation and quantification of molecular reorganization in PTFEMA bottlebrushes has been conducted by Ovchinnikova and her team. Their results were supported by theoretical simulations [164].

Ohe and his team incorporated complex bottlebrush architectures with PMA chains to accomplish targeted tumor imaging [72,73]. Kasi, Wei and their teams employed bottlebrushes containing PAA for induced intrafibrillar mineralization of collagen fibrils [97]. Wooley and her team synthesized fluoro-containing molecular brush copolymers and tested them as lithographic materials. Large areas of vertical alignment of the cylindrical bottlebrushes in thin films allowed near molecular pixel resolution after electron beam lithography [114,115,165]. Tang and his team reported oligothiophene-containing polymer brushes and their application as nanodielectric materials in capacitors. Polymer brush architecture was proven to be an effective method to improve the mechanical robustness of films [166]. PMMA bottlebrushes were used as part of an electrode interlayer in organic solar cell devices leading to enhanced device performance by Qiao and his colleagues [162]. A TEMPO containing side polymeric chain bottlebrush copolymer was synthesized by Nishide and his coworkers and tested as electrode-active materials in redox flow batteries [167]. Exceptionally large bottlebrush nanoparticles were considered for production of hybrid materials for many important applications by Watkins and his team [70,71]. Matson and his team investigated the adhesive properties of P*n*BA bottlebrushes, which were reversibly cross-linked [168].

### 6.6. Other Side Chains

In this section molecular brushes that bear side chains other than the aforementioned are listed. Some of the papers have already been mentioned since the molecular brushes contained PEO, PS, P(D)LLA, PCL, P(meth)acrylates too. They are mentioned here to provide useful information to the synthetic chemist mainly.

Thiophene-containing molecular brushes have appeared in 6 papers [139,143,166,169,170,171]. They were synthesized by “grafting through” the appropriate macromonomers in each case with **G3’** or by “grafting from” the backbone. Morphologic properties have been investigated in every paper by TEM, AFM, SEM, WAXD, etc. Their thermal properties have been studied as well. Light harvesting, optoelectronics, membrane separation and nanodielectric materials are some of the applications these polymers could be efficient to.

Bottlebrushes containing PDMS side chains have been synthesized in 6 cases [118,119,138,142,172]. Apart from the academic interest, their properties as lithographic materials have been studied.

Molecular brushes with N-phenyl maleimide side chains have been synthesized by Wooley and her colleagues in four cases [57,113,114,115]. The polymers were intended to be used as lithographic materials and morphology was the main property studied.

Poly(hexyl isocyanate) and poly(4-phenylbutyl isocyanate) have been incorporated in molecular brushes exhibiting photonic crystal properties [173]. Chiral block molecular brushes PLLA-*b*-PHIC, PLLA-*b*-PHIC-*b*-PLLA and PHIC-*b*-PLLA-*b*-PHIC have been synthesized by our team [50] and a core–shell structure with PLLA-*b*-PHIC side chains [148]. Their optical and thermal properties were investigated.

Bottlebrushes with NBE side chains have been synthesized using Mo catalysts by Nomura and his coworkers [28,29,41]. The DP was only 10 and no further investigation was done. The polymerization of NBE macromonomers with Grubbs’ catalysts has not been mentioned in the literature.

Poly(2-vinylpyridine)/PS pseudo-alternating [119] and poly(4-vinylpyridine)-*b*-PS diblock [108] molecular brushes have been synthesized. Only their synthesis is mentioned in the corresponding articles.

One article referring to bottlebrushes with poly(vinyl acetate) side chains has been published by Matson and his colleagues [174].

Molecular brushes with poly(N-isopropylacrylamide) (PNIPAAM) side chains has been mentioned in the literature in one case [60].

Bottlebrushes with poly(isobutylene) side chains have been synthesized recently [175]. The relative rates of oxanorbornene and norbornene macromonomers revealed the faster polymerization with the latter end groups.

Poly(phenyl isocyanide) side chains have been grown from the norbornene backbone by Wu and his team. “Grafting through” the appropriate macromonomers was proven impossible as the authors mention. The bottlebrushes exhibited worm-like morphology [176].

In 2001, Allcock and his colleagues “grafted through” polyphosphazene macromonomers. Their main purpose was to copolymerize the macromonomer with small monomers, but molecular brushes were also prepared using **G1** [177].

## 7. Summary

In the last decade, polymer brushes prepared by ROMP of NBE macromonomers have acknowledged great interest from various research teams and more than 100 papers have been published. Since polymerizing macromonomers by the “graft through” method is the best way to synthesize molecular brushes, Grubbs’ third generation catalyst provided the required tool to polymerize macromonomers bearing a variety of functional groups in minimum reaction time. The living character of **G3** provided the means towards controlled polymerization reactions leading to well-characterized bottlebrushes with narrow polydispersities. This feature enabled the design and synthesis of advanced structures containing bottlebrush segments for special applications.

Drug and gene delivery, tissue engineering scaffolding and tumor imaging are some of the fields where molecular brushes have been studied. Materials for photonic crystals, liquid crystals, batteries, solar cells, lithography have also been synthesized and studied.

## Abbreviations

P2VP	poly(2-vinyl pyridine)
P4VP	poly(4-vinyl pyridine)
P3HT	poly(3-hexyl thiophene)
PBd	polybutadiene
PCL	poly(ε-caprolactone)
PDLA	poly(d-lactide)
PDLLA	poly(d,l-lactide)
PDMAEMA	poly(2-(dimethylamino)ethyl methacrylate)
PDMS	poly(dimethyl siloxane)
PE	polyethylene
PEG	poly(ethylene glycol)
PEO	poly(ethylene oxide)
PHIC	poly(hexyl isocyanate)
PLLA	poly(l-lactide)
PMan	poly(maleic anhydride)
PMMA	poly(methyl methacrylate)
P*n*BA	poly(*n*-butyl acrylate)
PNBE	polynorbornene
PNIPAAM	poly(*N*-isopropylacrylamide)
PBAEAM	P(*N*-*tert*-butyloxycarbonyl-*N*′-acryl-1,2-diaminoethane)
PS	polystyrene
PSOH or P*p*HS	poly(*p*-hydroxy styrene)
P(pHS-co-PhMI)	poly(*p*-hydroxystyrene-co-N-phenylmaleimide)
P*t*BA	poly(*tert*-butyl acrylate)
P*t*BOS	poly(*tert*-butoxy styrene)
PTFEMA	poly(2,2,2-trifluoroethyl methacrylate)
PTF*p*HS	poly(tetrafluoro-*p*-hydroxystyrene)
